# Processed silkworm powder (Hongjam) ameliorates metabolic dysfunction-associated steatotic liver disease via GPR35/PKA and SIRT1/AMPK pathways

**DOI:** 10.3389/fnut.2025.1727043

**Published:** 2025-12-03

**Authors:** Da-Young Lee, Hye-Rin Ahn, Young-Min Han, Moon-Young Song, Seung-Won Lee, You-Kyung Jang, Seokho Kim, Eun-Ji Go, Eun-Hee Kim

**Affiliations:** 1 College of Pharmacy and Institute of Pharmaceutical Sciences, CHA University, Seongnam, Republic of Korea; 2 QBM Co., Ltd., Seoul, Republic of Korea

**Keywords:** Hongjam, fibroin peptide, MASLD, lipogenesis, fatty acid oxidation, inflammation, gluconeogenesis

## Abstract

**Introduction:**

Metabolic dysfunction-associated steatotic liver disease (MASLD) is a chronic metabolic disorder linked to obesity, insulin resistance, and type 2 diabetes. This study aimed to investigate the preventive effects of Hongjam, a processed silkworm powder, in a high-fat diet–induced MASLD mouse model and to explore its underlying mechanisms.

**Methods:**

C57BL/6J mice were fed a high-fat diet with or without Hongjam supplementation. Serum lipid and glucose levels, hepatic lipid accumulation, liver injury markers, and inflammatory responses were evaluated. Mechanistic analyses focused on SIRT1/AMPK signaling, lipogenic and fatty acid oxidation gene expression, STAT3 phosphorylation, cytokine production, and GPR35/PKA-mediated gluconeogenesis. To identify bioactive components, a silk-derived fibroin peptide was synthesized and tested in palmitate-treated HepG2 cells.

**Results:**

Hongjam supplementation improved serum triglyceride, cholesterol, and glucose levels, reduced hepatic lipid accumulation, and lowered liver injury markers. These beneficial effects were associated with activation of the SIRT1/AMPK pathway, suppression of lipogenesis, and enhancement of fatty acid oxidation. Hongjam also attenuated hepatic inflammation by inhibiting STAT3 phosphorylation and reducing pro-inflammatory cytokines, while restoring gluconeogenic capacity through GPR35/PKA signaling. The fibroin peptide suppressed SREBP1 expression, reduced lipid accumulation, and reactivated SIRT1/AMPK and GPR35/PKA/CREB pathways in HepG2 cells.

**Discussion:**

These findings suggest that Hongjam and its fibroin-derived peptide exert protective metabolic effects by modulating lipid metabolism, inflammation, and glucose homeostasis. Together, they highlight the potential of Hongjam as a functional food ingredient for the prevention of MASLD.

## 1 Introduction

The global prevalence of metabolic dysfunction-associated steatotic liver disease (MASLD) was estimated at 1.66 billion cases by 2019, representing 30% of the total population (1). MASLD occurs due to the accumulation of triglycerides in hepatocytes and is closely associated with obesity, insulin resistance, and type 2 diabetes mellitus (2). Although MASLD is asymptomatic, if not detected early, it can progress to metabolic dysfunction-associated steatohepatitis (MASH), cirrhosis, and hepatocellular carcinoma, posing significant health risks (3). Recent studies have focused on developing MASLD treatments that target peroxisome proliferator-activated receptor and glucagon-like peptide-1 receptor to improve insulin resistance, thereby reducing fat accumulation and inflammation. Additionally, research has focused on improving lipid metabolism to inhibit fat accumulation and enhance fatty acid oxidation in the liver (4). The sirtuin1 (SIRT1)/AMP-activated protein kinase (AMPK) signaling pathway is crucial for regulating hepatic lipid metabolism, promoting free fatty acid (FFA) oxidation by phosphorylating and inactivating acetyl-CoA carboxylase (ACC) and reducing triglyceride accumulation by suppressing transcriptional regulators like sterol regulatory element-binding protein 1c (SREBP1c) (5). Inflammation, a key feature of fatty liver disease, is driven by the nuclear factor-κB (NF-κB) pathway, which regulates inflammatory cytokines like tumor necrosis factors-*α* (TNF-α), interleukin (IL)-1β, IL-6 (6, 7). Additionally, IL-6 activates the signal transducer and activator of transcription 3 (STAT3) pathway, exacerbating hepatic inflammation and worsening liver damage (7, 8). Therefore, effective management of fatty liver disease requires strategies that suppress both lipid accumulation and inflammation within hepatocytes.

Gluconeogenesis, the process of endogenous synthesis of glucose primarily in the liver, is essential for maintaining whole-body glucose homeostasis during fasting and increased energy demands (9). Dysregulation of gluconeogenesis is associated with metabolic disorders (10). In the context of MASLD, recent findings suggest that abnormal gluconeogenesis may contribute to the progression of MASLD to more serious liver diseases such as MASH and cirrhosis (10). Gluconeogenesis is regulated through allosteric and transcriptional activation of phosphoenolpyruvate carboxykinase 1 (PCK1) and glucose-6-phosphatase (G6Pase) (11). Additionally, protein kinase A (PKA), a cAMP-dependent protein kinase, promotes glycogen breakdown and thus gluconeogenesis (11). G protein-coupled receptors (GPCRs) have been reported as upstream regulators of PKA activity, and recently, G protein-coupled receptor 35 (GPR35), an orphan GPCR, has been attracting attention as a target for treating various diseases (12, 13). Abnormalities in the coding and intergenic regions of GPR35 have been reported to be associated with metabolic diseases such as type 2 diabetes, and activation of GPR35 has been shown to prevent lipid accumulation by increasing energy expenditure in adipose tissue (14–17). In fact, treatment with lodoxamide, a potent agonist of GPR35, has been reported to alleviate MASLD and MASH (3). Therefore, identification of the specific signaling pathway of GPR35 involved in MASLD and gluconeogenesis represents a new strategy for MASLD prevention.

Silkworms have traditionally been used as an ingredient in folk remedies for various health-promoting functions (18). They contain a large amount of protein, omega-3 fatty acids, vitamins, and minerals (19). As silkworm larvae grow, they produce a large amount of silk protein. After the 3rd day of the 5th instar, the larvae are filled with silk fibers, making them difficult to eat. However, with the recent development of a new technology that can steam, freeze-dry, and grind silkworms to make them edible, the potential for preventing or treating liver damage, Parkinson’s disease, and diabetic hyperglycemia has been reported, attracting scientific attention (20–23). Previously, we confirmed that Hongjam has a gastritis-preventing effect in rats with induced alcoholic gastritis (24). Furthermore, it was established that Hongjam prevents fatty liver disease in rats with induced alcoholic fatty liver disease (25). These findings highlight the importance of exploring edible insect-derived products such as Hongjam for their potential as functional food ingredients targeting liver health.

Hongjam (50 mg/kg) was found to prevent alcoholic fatty liver disease in rats by increasing phosphorylation of AMPK, thereby reducing lipogenesis, and increasing FFA oxidation by increasing phosphorylation of ACC. In addition, the possibility of reducing alcoholic liver inflammation was confirmed by reducing the expression of pro-inflammatory cytokines such as tumor necrosis factor-*α* and interleukin-1β (25). Despite these promising findings, the protective effects of Hongjam against MASLD have not yet been reported. In this study, for the first time, we examined whether fibroin peptide, one of the major proteins derived from silkworms, exerts beneficial effects on hepatic lipid metabolism and gluconeogenesis *in vitro* model, alongside the *in vivo* evaluation of Hongjam in HFD-induced MASLD model. Considering the growing consumer demand for functional foods with metabolic health benefits, identifying natural ingredients that can ameliorate MASLD is of great interest to food scientists and industry.

## 2 Materials and methods

### 2.1 Preparation of Hongjam

Hongjam was made as previously described (20). Briefly, *Bombyx mori* white-jade cocoon strain, 7th day of 5th instar, was steamed at 100 °C for 130 min using an electric pressure-free cooking machine (KumSeong Ltd., Bucheon, Korea). Then, the samples were freeze-dried for 24 h using a non-pressure cooker and freeze dryer (FDT-8612, Operon Ltd., Kimpo, Korea). Subsequently, they were ground to a particle size of less than 0.1 mm using a disk mill (HM001, Korean Pulverizing Machinery Co. Ltd., Incheon, Korea). Then, Hongjam was stored at −50 °C for the experiment.

### 2.2 High-performance liquid chromatography chromatogram of Hongjam

To confirm the chromatographic profile of Hongjam, hydrochloric acid containing Hongjam, amino acids, and 0.05% mercaptoethanol were added. After removing hydrochloric acid by repeated drying at 40 °C, the residue was dissolved in sodium citrate buffer or hydrochloric acid solution and filtered through a membrane filter prior to analysis. Amino acid profiling was performed using an Amino acid analyzer L-8900 (Hitachi High-Technologies Corporation, Tokyo, Japan). Chromatographic separation was achieved on a Na-type ion-exchange resin column [Main column: 855–4,506 (4B6813), 60 mm × 4.6 mm; Guard column: 855–6,268 (160372); Ammonia filter column: 855–3,523 (D8683); all from Hitachi High-Technologies]. The column oven temperature was maintained at 57 °C, and the injection volume was 10 μL. The mobile phases consisted of the manufacturer-supplied buffers for ion-exchange amino acid analysis, including PH-1, PH-2, PH-3, PH-4, H_2_O, and PH-RG. Separation was performed using a gradient elution program automatically controlled by the instrument: 0.00–7.30 min (100% PH-1); 7.30–20.60 min (100% PH-2); 20.60–28.80 min (100% PH-3); 28.80–35.40 min (100% PH-4); 35.40–36.20 min (100% H_2_O); 36.20–39.10 min (100% PH-1), followed by column regeneration according to the standard L-8900 protocol. The flow rate was 0.40 mL/min during separation and 0.35 mL/min during regeneration. Post-column derivatization with ninhydrin was performed at a reactor temperature of 135 °C, and detection was carried out at 570 nm.

### 2.3 Peptide synthesis and preparation

The fibroin peptide used in this study was custom-synthesized by Peptron (Daejeon, Korea). The sequence Cys-Gly-Ala-Gly-Ala-Gly-Ser-Asn (C-GAGAGS-N) was synthesized via solid-phase peptide synthesis using a PeptrEX automated synthesizer (Peptron, Daejeon, Korea). Lyophilized peptides were dissolved in sterile distilled water immediately before use. Analytical HPLC confirmed a purity of ≥ 98% (Supplementary Figure 2).

### 2.4 Cell culture

Human hepatocellular carcinoma HepG2 cells (ATCC HB-8065; RRID: CVCL_0027) were obtained from the American Type Culture Collection (ATCC; Rockville, MD, United States) and were cultured in Dulbecco’s modified Eagle medium (DMEM; Gibco, Waltham, United States) supplemented with 10% fetal bovine serum (FBS; Gibco, Waltham, United States) and 100 U/mL penicillin and 100 μg/mL streptomycin (Pen-Strep; Gibco, Waltham, United States) at 37 °C in a 5% CO_2_ incubator. HepG2 cells were treated with 200 μM palmitic acid (PA) for 48 h to model metabolic dysfunction-associated steatotic liver disease (MASLD). PA was prepared by dissolving sodium palmitate (Sigma-Aldrich, St. Louis, United States) in fatty acid free bovine serum albumin (BSA; Sigma-Aldrich, St. Louis, United States) solution in serum-free DMEM to yield a 5 mM stock. Vehicle control cells were treated with an equivalent volume of the BSA solution.

### 2.5 Animal experiments

All animal experiment procedures were conducted in accordance with the guidelines and approval of the Institutional Animal Care and Use Committees (IACUC) of the CHA University (reference number: 220115). Five-week-old male C57BL/6 N mice were obtained from Orient Bio (Gyeonggi-do, Korea) and housed in a pathogen-free facility at room temperature with a 12-h light/dark cycle. The mice were allowed an acclimatization period of 1 week, during which they were fed a commercial diet (Haran 2018s) with ad libitum access to tap water. Thereafter, the mice were divided into five groups (*n* = 8/group), which were each allocated to one of five experimental diets: normal diet, high-fat diet (HFD), and HFD supplemented with 0.01 g/kg Hongjam, 0.1 g/kg Hongjam, and 0.1 g/kg silymarin (S0292, Sigma-Aldrich), respectively. The mice were subjected to the experimental diet for 12 weeks, during which time all animals were permitted ad libitum access to the diet and water. The composition of the diets used in the experiment is shown in Supplementary Table 1. Throughout the experimental period, weekly body weight and food intake were measured. At the end of the experiment, mice were anaesthetized with carbon dioxide inhalation. Blood was drawn from the abdominal aorta into a heparin-coated tube. Plasma was subsequently obtained by centrifuging the blood at 5,000 rpm for 30 min at 4 °C. The livers were excised, rinsed with phosphate-buffered saline (PBS), and weighed. A portion of each liver was fixed in 10% formalin for further analysis. The plasma and liver samples were stored at −80 °C until analysis.

### 2.6 Serum analysis and ELISA

The serum levels of triglyceride, glucose, total cholesterol, low density lipoprotein (LDL)-cholesterol, high density lipoprotein (HDL)-cholesterol, alanine aminotransferase (ALT), aspartate aminotransferase (AST), gamma glutamyl transpeptidase (GGT), and bilirubin were analyzed using Hitachi automatic analyzer 7,600–210 (Hitachi High-Technologies Corporation, Tokyo, Japan). The serum concentration of tumor necrosis factor-alpha (TNF-*α*) and interleukin-1 beta (IL-1β) were measured by using a commercial mouse ELISA kit (R&D System, Minneapolis, MN, United States) following manufacturer’s protocol.

### 2.7 Hepatic triglyceride analysis

For the measurement of triglyceride levels in liver tissues, we used the commercial triglyceride assay kit (ab65336, Abcam, Cambridge, United Kingdom). The liver (0.1 g) washed with PBS was homogenized in 5% NP-40 solution. Next, the sample was slowly heated at 100 °C for 5 min, then cooled to room temperature, which was repeated twice. After that, the sample was centrifuged at 15,000 rpm for 2 min, and the supernatant was collected and analyzed according to the manufacturer’s instructions.

### 2.8 Glycogen assay

Hepatic glycogen concentration was measured using a commercial Glycogen assay kit (ab65620, Abcam, Cambridge, United Kingdom) according to the manufacturer’s protocol. Briefly, approximately 100 mg of frozen liver tissue was resuspended in 30% KOH. The samples were heated at 100 °C for 2 h to digest tissue and solubilize glycogen. After cooling, two volumes of 95% ethanol were added to precipitate crude glycogen. The mixture was centrifuged at 18,000 × g for 10 min at 4 °C, and the supernatant was discarded. The precipitate was resuspended in a minimal volume of double-distilled water and acidified to pH 3 by adding 5 N HCl dropwise. 95% ethanol was then added to re-precipitate glycogen. The final pellet was washed in 95% ethanol, air-dried, and dissolved in assay buffer 8 for colorimetric measurement. For quantification, 50 μL of the dissolved sample was incubated with hydrolysis enzyme mix I and reaction mix at room temperature for 30 min in the dark, and absorbance was read at 570 nm using a microplate reader (SpectraMax iD3, Molecular Devices, United States). Glycogen concentrations were calculated from a standard curve and normalized to tissue weight.

### 2.9 Histological analysis

Formalin-fixed liver samples were embedded in paraffin, sliced at 5 μm, followed by sectioning and hematoxylin and eosin (H&E) staining by standard procedures. Then, H&E stained samples were visualized using a Leica microsystem (Leica DM750, Wetzlar, Germany). Histopathological scoring was assessed by an experienced pathologist, who was blinded to the treatment groups. Levels of fatty infiltration and steatosis were graded as 0 point for no hepatocytes affected, 0.5 point for slightly affected (0–5%), 1 point for mildly (5–20%), 2 points for moderately (20–50%), and 3 points for severely (>50%) (26).

### 2.10 Oil-red O staining

The frozen tissue sections were soaked in 60% isopropyl alcohol for 20–30s and then stained with ORO staining solution (O1391, Sigma-Aldrich) for 15 min at room temperature. Then, the sections were washed again with 60% isopropanol and distilled water to remove non-specific binding stains. The degree of ORO staining was scored as follows: No staining = 0; < 30% staining = 1; < 60% staining = 2; > 90% staining = 3.

### 2.11 RNA isolation and gene expression analysis

Total mRNA was isolated from the mouse livers using Trizol reagent (Invitrogen, Carlsbad, CA, United States) and cDNA was synthesized using SuperScript® II Reverse Transcriptase kit (Invitrogen, Carlsbad, CA, United States) according to the manufacturer’s instructions. The mRNA levels were analyzed by quantitative real-time polymerase chain reaction (qRT-PCR). The qRT-PCR was assessed as previously reported (27) and was performed on a ViiATM 7 real-time PCR system (Life Technologies Corporation, Carlsbad, CA, United States) using Luna universal qPCR master mix (New England Biolabs, Beverly, MA, United States). The genes were amplified 20 s at 95 °C and then 40 cycles at 95 °C (3 s), 60 °C (30 s) followed by melting curve analysis. The primers used for the qRT-PCR were listed in Supplementary Table 2.

### 2.12 Western blotting

Liver tissues were homogenized with ice-cold cell lysis buffer containing protease inhibitor (Roche Applied Science, Mannheim, Germany) and samples were incubated on ice with frequent vortexing for 5 min and centrifuged for 15 min at 13,000 rpm. The protein concentration of each supernatant was quantified using PierceTM BCA protein assay kit (Thermo Fisher Scientific, Waltham, MA, United States) in accordance with the manufacturer’s instructions. The proteins were loaded onto a 10% sodium dodecyl sulfate polyacrylamide gel electrophoresis (SDS-PAGE), and transferred to polyvinylidene fluoride membranes (Millipore, Burlington, MA, United States). After transfer, membranes were blocked with 3% bovine serum albumin (BSA) in Tris-buffered saline with 0.05% Tween 20 (TBS-T) and probed with the specified primary antibodies (diluted 1:1000) overnight at 4 °C. The membranes were washed and incubated with the appropriate secondary antibodies in TBS-T for 1 h. The blots were then developed using an enhanced chemiluminescence system (Thermo Fisher Scientific, Waltham, MA, United States). Antibodies for p-AMPK, AMPK, SIRT1, CPT-1, PKA, CREB, PGC-1α, SREBP1, mTOR, and *β*-actin were purchased from Santa Cruz Biotechnology (Santa Cruz, CA, United States). Antibodies for p-ACC, ACC, p-STAT3, and STAT3 were purchased from Cell Signaling Technology (Danvers, MA, United States). Antibody for GPR35 was purchased from Proteintech (Manchester, England).

### 2.13 Statistical analysis

All values are expressed as mean ± SD. Statistical analysis was performed using one-way ANOVA and analyzed further by Tukey’s *post-hoc* test. Differences among experimental groups were considered to be statistically significant at *p* < 0.05. All statistical analyses were performed with GraphPad Prism software (San Diego, CA, United States).

## 3 Results

### 3.1 HPLC chromatogram analysis of Hongjam

**Figure 1:**
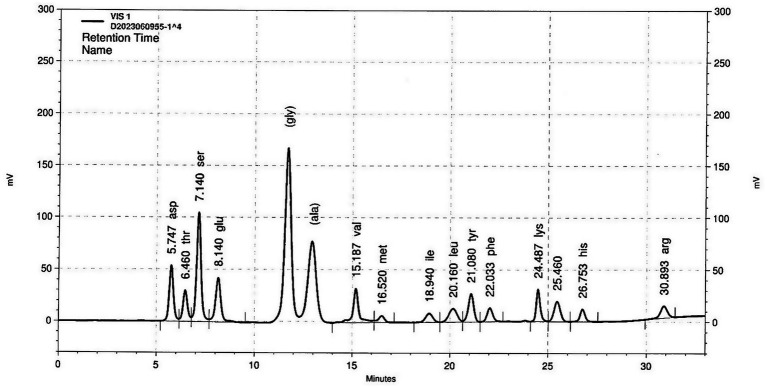
HPLC chromatogram analysis of Hongjam.

The purity of Hongjam was assessed using HPLC analysis, which revealed that aspartic acid (Asp), threonine (Thr), serine (Ser), glutamic acid (Glu), glycine (Gly), alanine (Ala), valine (Val), methionine (Met), isoleucine (Ile), leucine (Leu), tyrosine (Tyr), phenylalanine (Phe), lysine (Lys), histidine (His), and arginine (Arg) were the major components of Hongjam (Figure 1). Among these amino acids, the most abundant was Gly, followed by Ser and Ala (Figure 1).

### 3.2 Hongjam alleviates MASLD in HFD-fed mice

**Figure 2:**
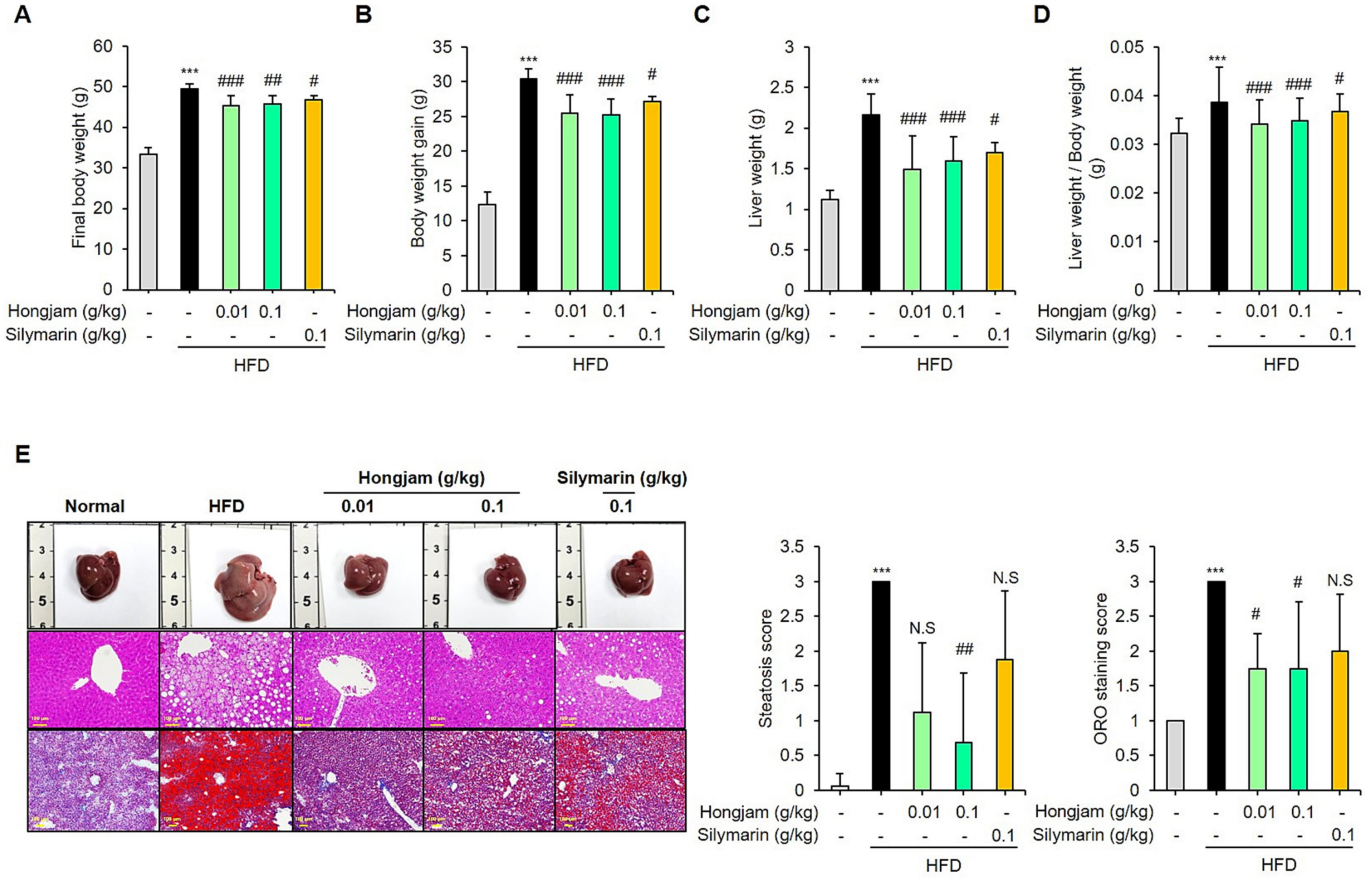
Hongjam alleviates metabolic dysfunction-associated steatotic liver disease (MASLD) in HFD-fed mice. Six-week-old male C57BL/6 N mice (*n* = 8 per group) were randomly divided into five groups: normal diet, HFD, HFD + Hongjam (0.01 g/kg), HFD + Hongjam (0.1 g/kg), HFD + silymarin (0.1 g/kg). After 12 weeks, **(A)** changes in body weight, **(B)** body weight gain, **(C)** liver weight, and **(D)** liver weight/body weight ratio were measured. **(E)** Representative pictures of livers, H&E staining, ORO staining for liver (scale bar = 100 μm), and hepatic steatosis and ORO staining scores. Data are expressed as mean ± SD (*n* = 8). Statistical analysis was performed using one-way ANOVA followed by Tukey’s *post–hoc* test. *p < 0.05, **p < 0.01, and ***p < 0.001 vs. normal group; #*p* < 0.05, ##*p* < 0.01 and ###*p* < 0.001 vs. HFD group.

Hongjam administration for 12 weeks significantly decreased the body weight in mice fed HFD (Figure 2A). After 12 weeks of treatment, Hongjam reduced the HFD-induced weight gain in mice (Figure 2B). The mass of liver tissue from mice fed the HFD supplemented with Hongjam was lower than those fed the HFD only (Figure 2C). Hongjam group, as compared with HFD group, had a reduction in liver weight to body weight ratio (Figure 2D). Hongjam-fed mice displayed lower gross liver weight compared with HFD-fed mice (Figure 2E). Liver H&E and ORO staining data showed that the remarkable lipid droplets appeared in the livers of HFD-fed mice were effectively diminished upon Hongjam administration (Figure 2E). To confirm these findings, steatosis and ORO staining scores were evaluated in H&E and ORO staining images of liver tissues from normal diet-, HFD-, and Hongjam-fed mice. The HFD-induced elevation of the hepatic steatosis score was significantly normalized in the livers of Hongjam-fed mice. Silymarin administration also reduced body weight gain, liver weight, and ORO-stained lipid accumulation, but Hongjam was more effective (Figure 2).

### 3.3 Hongjam attenuates MASLD and liver injury in HFD-fed mice

**Figure 3:**
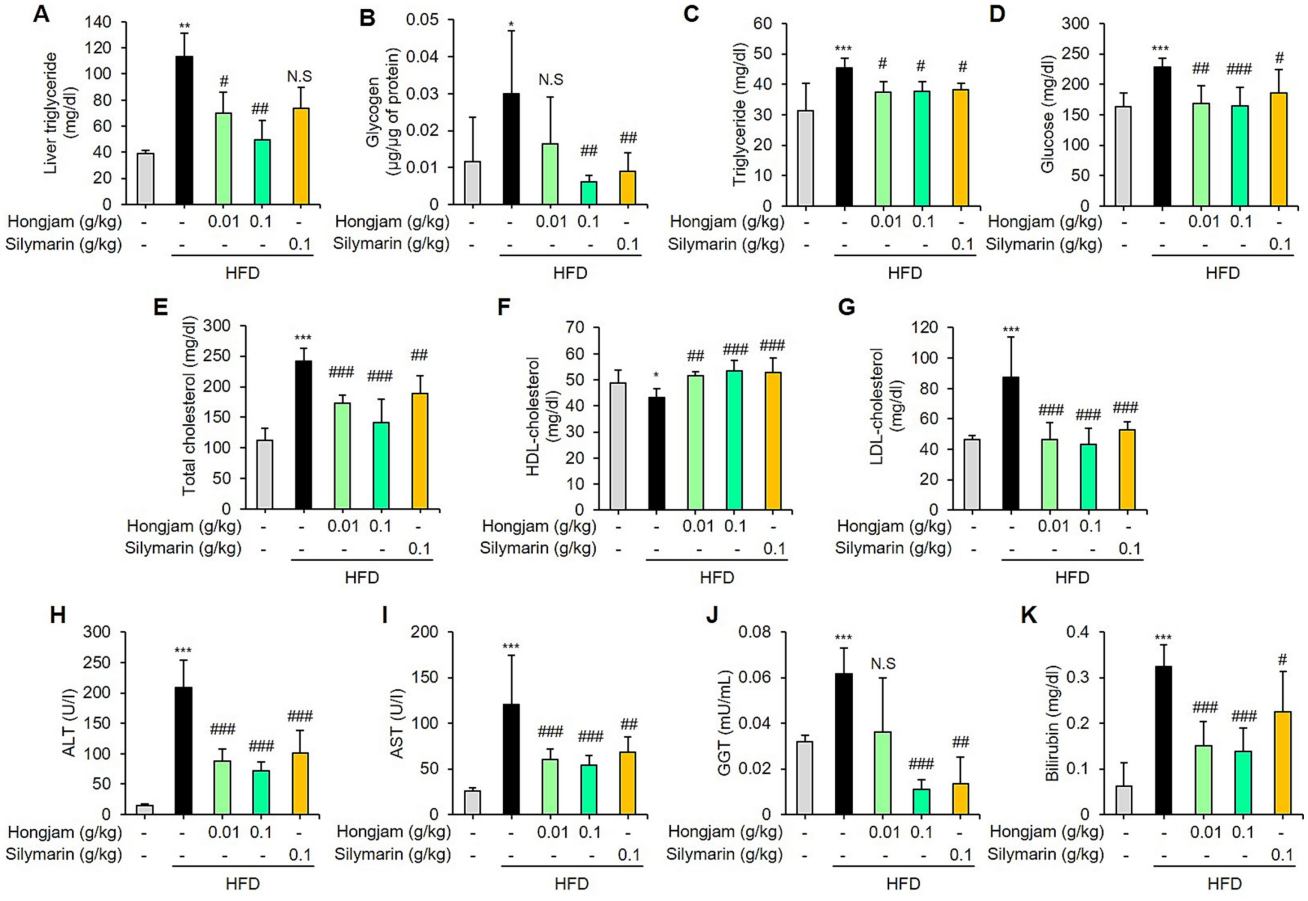
Hongjam attenuates MASLD and liver injury in HFD-fed mice. Six-week-old male C57BL/6 N mice (*n* = 8 per group) were randomly divided into five groups: normal diet, HFD, HFD + Hongjam (0.01 g/kg), HFD + Hongjam (0.1 g/kg), HFD + silymarin (0.1 g/kg). After 12 weeks, hepatic and plasma biochemical parameters were analyzed. **(A)** Liver triglyceride, **(B)** glycogen, plasma levels of **(C)** triglyceride, **(D)** glucose, **(E)** total cholesterol, **(F)** LDL-cholesterol, **(G)** HDL-cholesterol were measured. Plasma levels of **(H)** ALT, **(I)** AST, **(J)** GGT, and **(K)** bilirubin were measured. Data are expressed as mean ± SD (*n* = 8). Statistical analysis was performed using one-way ANOVA followed by Tukey’s *post–hoc* test. *p < 0.05, **p < 0.01, and ***p < 0.001 vs. normal group; #*p* < 0.05, ##*p* < 0.01 and ###*p* < 0.001 vs. HFD group.

We performed biochemical analysis to confirm the efficacy of Hongjam in suppressing fatty liver disease. HFD feeding for 12 weeks successfully induced fatty liver and liver injury in mice, which were manifested by significant increase in hepatic triglyceride, glycogen, plasma triglyceride concentration, glucose, total cholesterol, LDL-cholesterol, ALT, AST, GGT and bilirubin levels compared with those of normal diet-fed mice (Figures 3A–K). Hongjam significantly reversed the HFD-induced hepatic accumulation of triglycerides (Figure 3A), as well as lowering the plasma triglyceride levels (Figure 3B). Moreover, Hongjam significantly reduced the HFD-induced glucose, total cholesterol, LDL-cholesterol, ALT, AST, GGT, and bilirubin levels (Figures 3C–J).

### 3.4 Hongjam activates SIRT1/AMPK-mediated signaling cascades in HFD-fed mice

**Figure 4:**
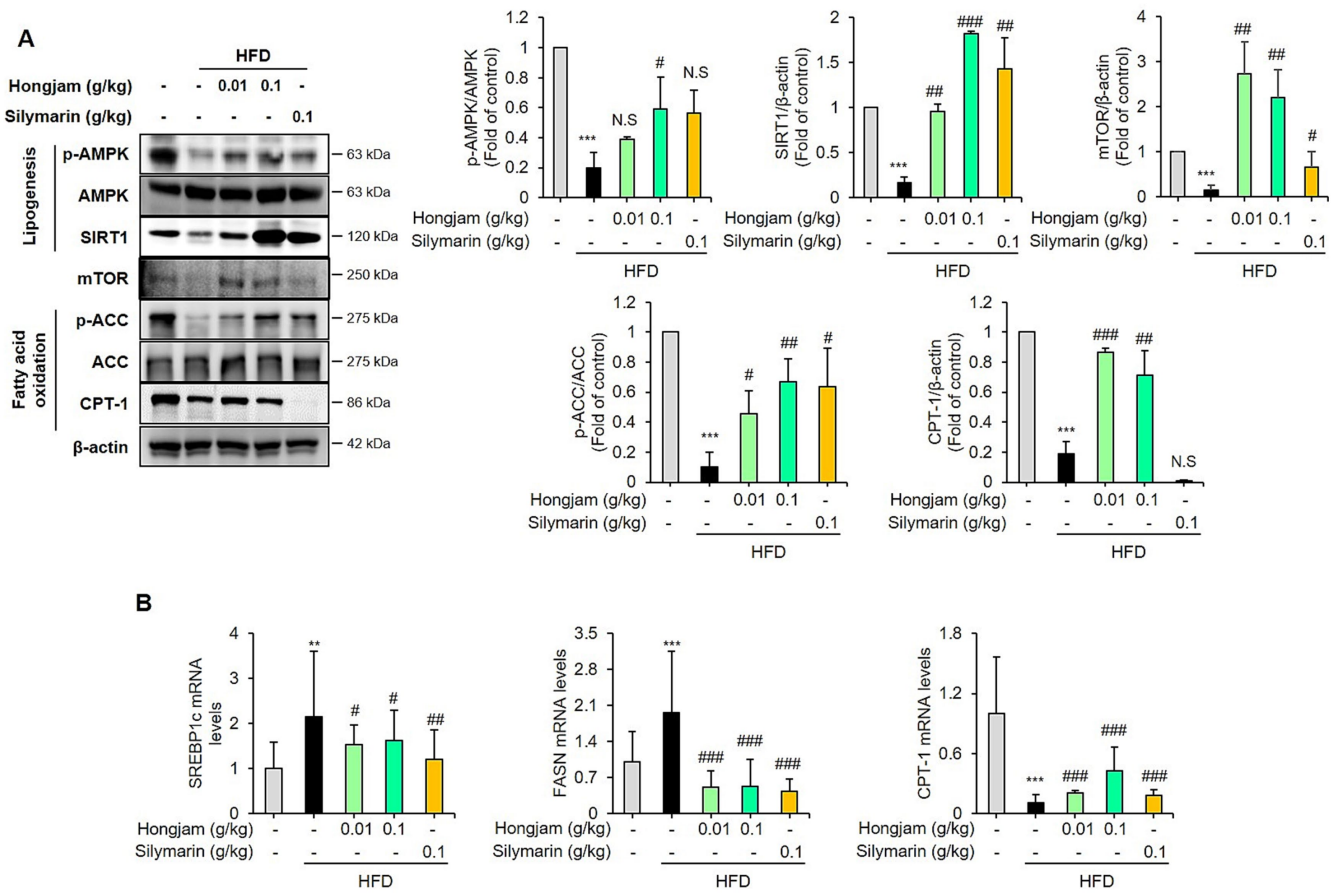
Hongjam activates SIRT1/AMPK-mediated signaling cascades in HFD-fed mice. Six-week-old male C57BL/6 N mice (*n* = 8 per group) were randomly divided into five groups: normal diet, HFD, HFD + Hongjam (0.01 g/kg), HFD + Hongjam (0.1 g/kg), HFD + silymarin (0.1 g/kg). After 12 weeks, hepatic signaling proteins and mRNA levels were analyzed. **(A)** Representative Western blot images and quantitative comparison of p-AMPK, SIRT1, p-ACC, CPT-1, and mTOR levels in livers from different groups, normalized by corresponding total AMPK, ACC, and *β*-actin. **(B)** Quantitative comparison of mRNA expression of SREBP1c, FASN, and CPT-1. All mRNA levels were normalized relative to Rn18S. Data are expressed as mean ± SD (*n* = 3). Statistical analysis was performed using one-way ANOVA followed by Tukey’s *post–hoc* test. *p < 0.05, **p < 0.01, and ***p < 0.001 vs. normal group; #*p* < 0.05, ##*p* < 0.01 and ###*p* < 0.001 vs. HFD group.

Western blotting results confirmed that Hongjam administration significantly increased the hepatic protein levels of p-AMPK, SIRT1, p-ACC, CPT-1, and mTOR (Figure 4A). Additionally, Hongjam administration significantly reversed the HFD-induced upregulation of hepatic SREBP1c and FASN mRNA (Figure 4B). The mRNA expression of CPT-1, regulators of fatty acid oxidation, was significantly upregulated in the livers of Hongjam-fed mice compared to the HFD mice (Figure 4B). Silymarin administration also effectively reduced lipogenesis through the upregulation of SIRT1/AMPK, however Hongjam administration proved to be more effective. In particular, Hongjam was more effective than silymarin in increasing FFA oxidation. These results suggest that Hongjam inhibits triglyceride accumulation and enhances fatty acid oxidation by activating the SIRT1/AMPK signaling pathway in the liver of mice fed a high-fat diet.

### 3.5 Hongjam reduces liver inflammation in HFD-fed mice

**Figure 5:**
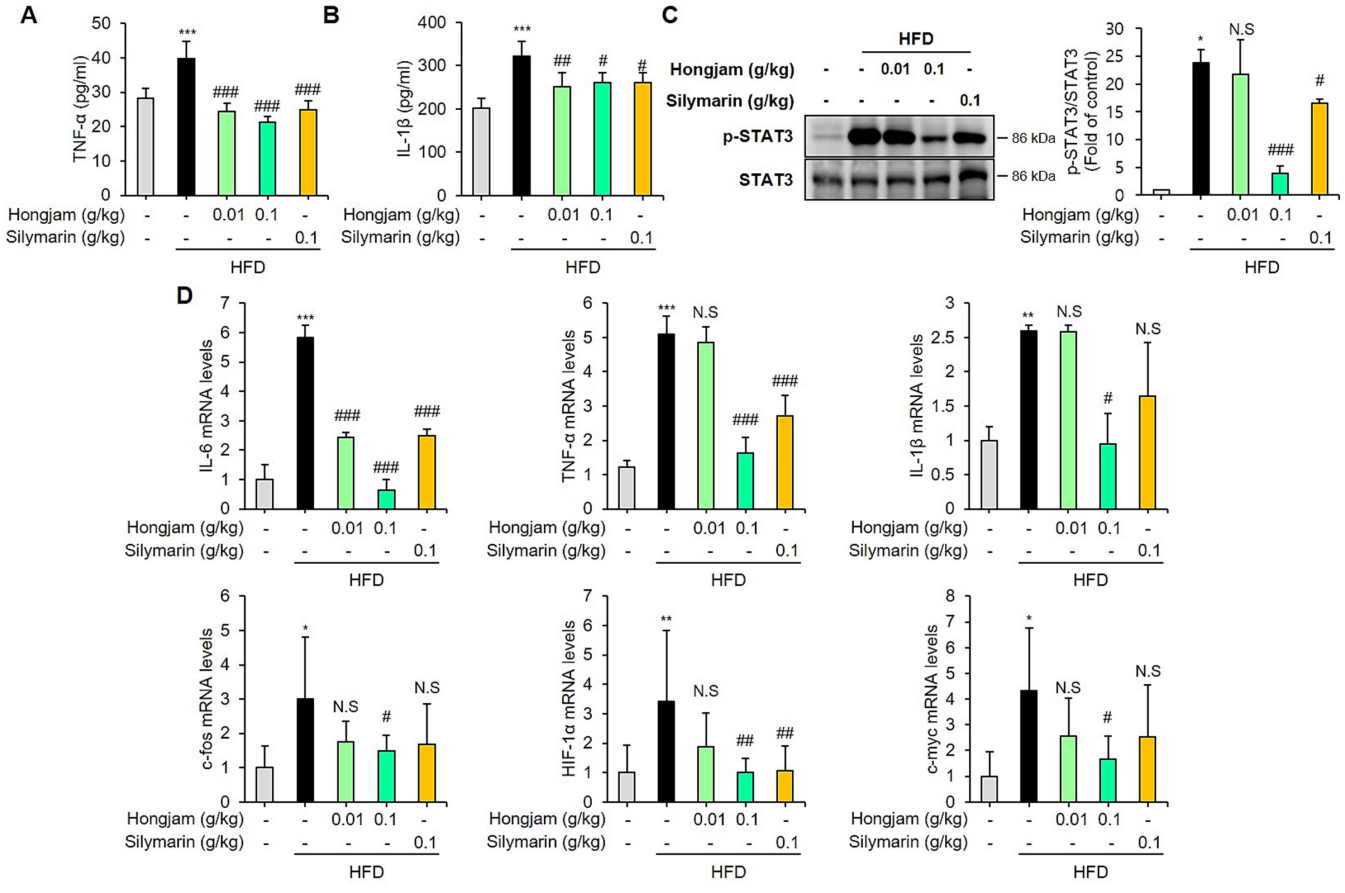
Hongjam reduces liver inflammation in HFD-fed mice. Six-week-old male C57BL/6 N mice (*n* = 8 per group) were randomly divided into five groups: normal diet, HFD, HFD + Hongjam (0.01 g/kg), HFD + Hongjam (0.1 g/kg), HFD + silymarin (0.1 g/kg). After 12 weeks, plasma levels of **(A)** TNF-α, and **(B)** IL-1β were measured. **(C)** Representative Western blots and quantitative comparison of p-STAT3 levels in liver, normalized by corresponding total STAT3. **(D)** Quantitative comparison of mRNA expression of IL-6, TNF-α, IL-1β, c-fos, HIF-1α, and c-myc. All mRNA levels were normalized relative to Rn18S. Data are expressed as mean ± SD (*n* = 3). Statistical analysis was performed using one-way ANOVA followed by Tukey’s *post–hoc* test. *p < 0.05, **p < 0.01, and ***p < 0.001 vs. normal group; #*p* < 0.05, ##*p* < 0.01 and ###*p* < 0.001 vs. HFD group.

The results showed a significant increase in these levels in HFD-fed mice, while Hongjam treatment decreased them (Figures 5A,B). As shown in Figure 5C, HFD-fed group induced the hepatic phosphorylation of STAT3, which was decreased dose-dependently by Hongjam treatment. Moreover, we performed qRT-PCR to determine the mRNA expression of pro-inflammatory cytokines such as IL-6, TNF-*α* and IL-1β. The expression of IL-6, TNF-α and IL-1β mRNA was significantly induced in the HFD group. However, Hongjam administration significantly reduced these pro-inflammatory cytokines (Figure 5D). We also confirmed the mRNA levels of STAT3 target genes in the liver. Treatment with HFD significantly induced the hepatic expression levels of c-fos, HIF-1α, and c-myc, while Hongjam intake significantly decreased the hepatic expression of c-fos, HIF-1α and c-myc (Figure 5D). Although silymarin administration was shown to reduce HFD-induced inflammation by inhibiting the phosphorylation of STAT3, Hongjam was found to be more effective. These results suggest that the consumption of Hongjam inhibits the STAT3 signaling pathway and reduces inflammatory responses in the liver of mice fed a high-fat diet.

### 3.6 Hongjam restores gluconeogenesis in HFD-fed mice

**Figure 6:**
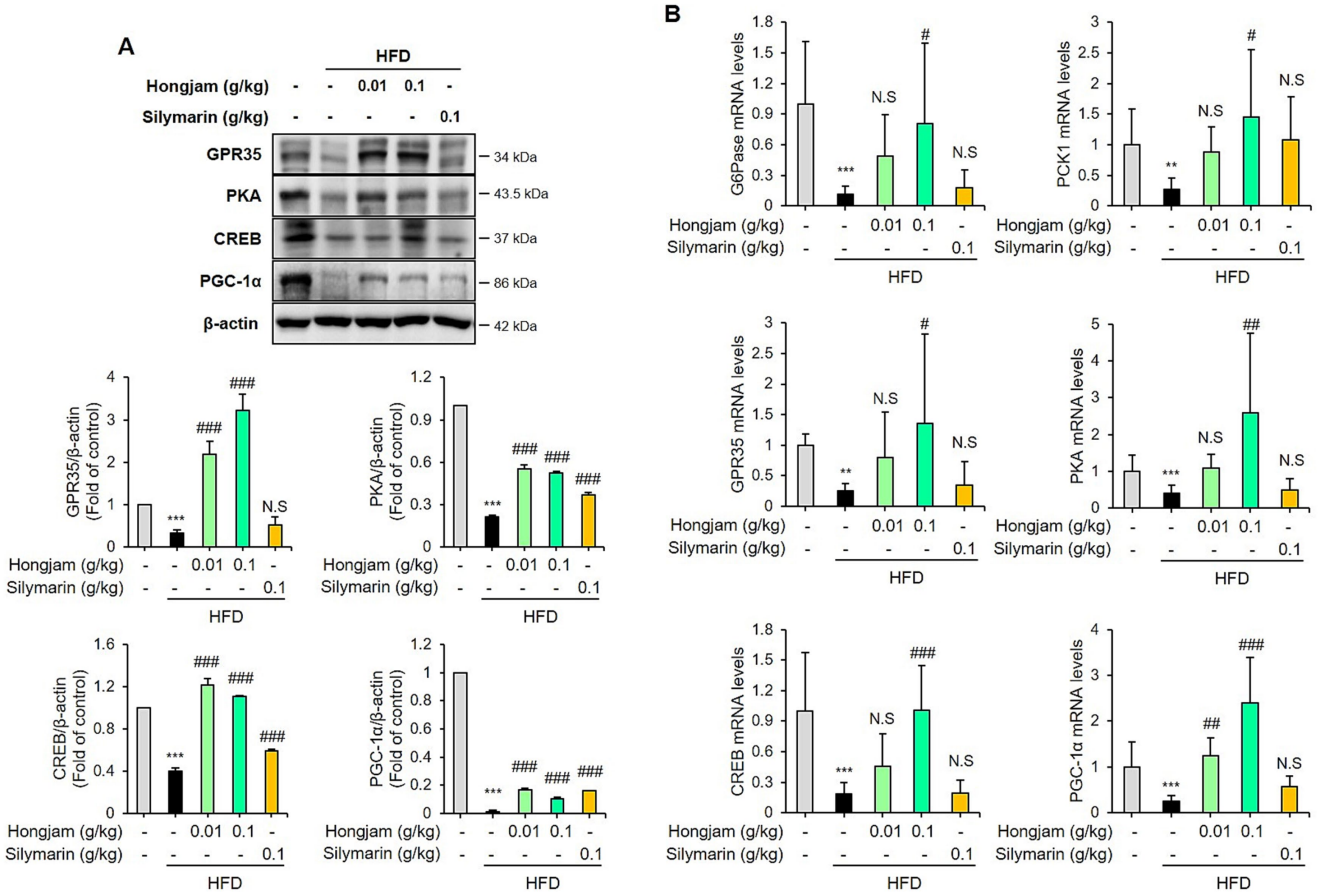
Hongjam restores gluconeogenesis in HFD-fed mice. Six-week-old male C57BL/6 N mice (*n* = 8 per group) were randomly divided into five groups: normal diet, HFD, HFD + Hongjam (0.01 g/kg), HFD + Hongjam (0.1 g/kg), HFD + silymarin (0.1 g/kg). After 12 weeks, hepatic protein and mRNA levels related to gluconeogenesis were analyzed. **(A)** Representative Western blot images and quantitative comparison of GPR35, PKA, CREB, and PGC-1α levels in liver from different groups, normalized by β-actin. **(B)** Quantitative comparison of mRNA expression of G6Pase, PCK1, GPR35, PKA, CREB, and PGC-1α. All mRNA levels were normalized relative to Rn18S. Data are expressed as mean ± SD (*n* = 3). Statistical analysis was performed using one-way ANOVA followed by Tukey’s *post-hoc* test. *p < 0.05, **p < 0.01, and ***p < 0.001 vs. normal group; #*p* < 0.05, ##*p* < 0.01 and ###*p* < 0.001 vs. HFD group.

Western blotting results confirmed that Hongjam administration significantly increased the hepatic protein expression of GPR35 (Figure 6A). Additionally, we confirmed the expression of PKA, which suppresses lipid accumulation as a downstream factor of GPR35 (28). As a result, Western blotting confirmed that Hongjam administration significantly increased the hepatic protein levels of PKA, CREB, and PGC-1α (Figure 6A). Next, we performed qRT-PCR to examine the expression of mRNA related to the GPR35 signaling pathway and gluconeogenesis. HFD administration significantly reduced the hepatic expression levels of GPR35 and PKA, and gluconeogenesis-associated genes such as CREB, PGC-1α, G6Pase, and PCK1. However, Hongjam administration significantly restored the hepatic expression levels of GPR35 and PKA, and gluconeogenesis-related genes such as CREB, PGC-1α, G6Pase, and PCK1, which had been reduced by HFD (Figure 6B). While silymarin administration did not effectively regulate the GPR35/PKA pathway, Hongjam administration effectively increased the expression of GPR35/PKA, thereby restoring gluconeogenesis, which had been reduced in the HFD group. These findings suggest that Hongjam alleviates MASLD by effectively increasing gluconeogenesis via the GPR35/PKA pathway.

### 3.7 Silk fibroin improves hepatic lipid metabolism and gluconeogenesis in PA-induced MASLD

**Figure 7:**
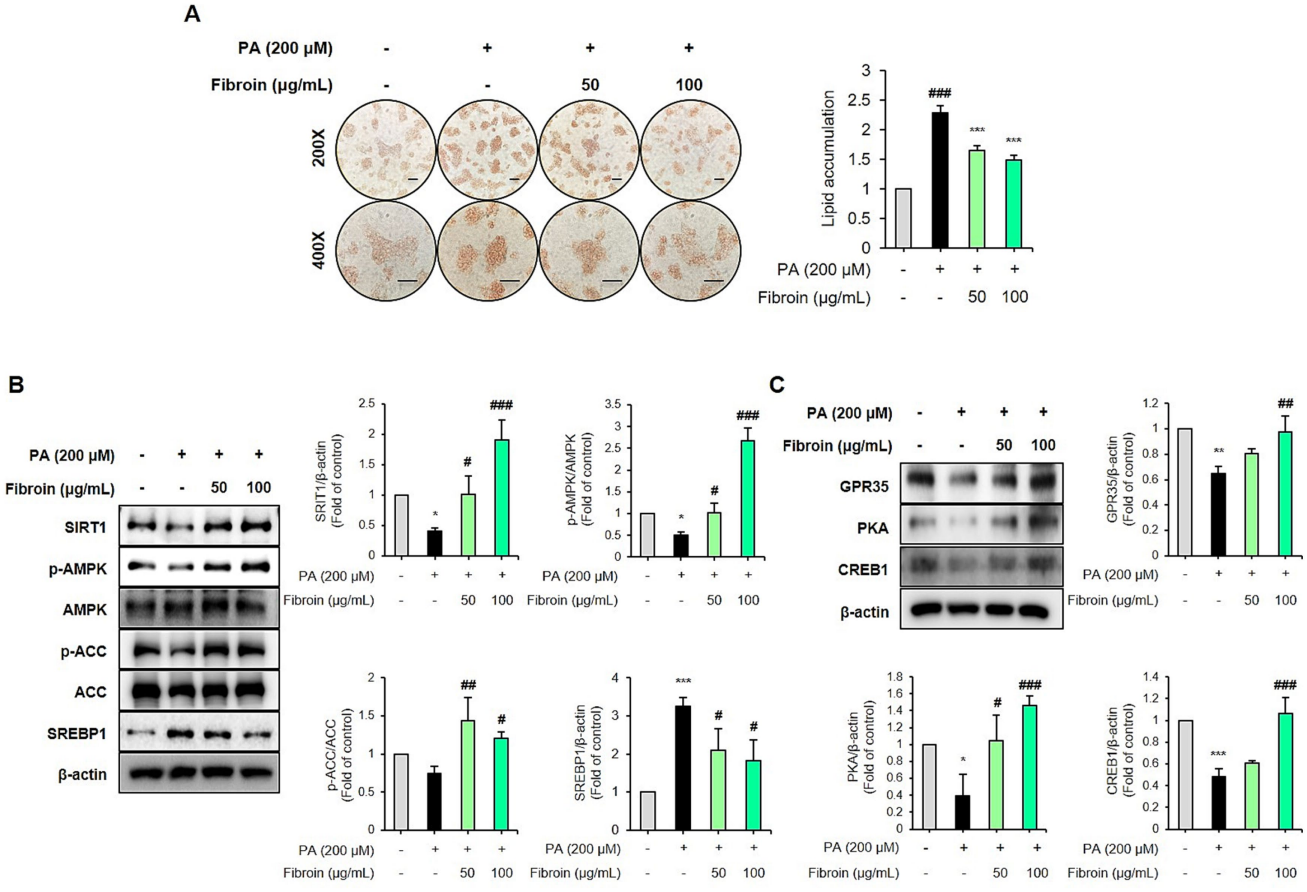
Silk fibroin peptide ameliorates lipid metabolism in palmitic acid (PA)-induced HepG2 cells. HepG2 cells were treated with 200 μM PA to induce lipid accumulation and co-treated with silk fibroin peptide (50 or 100 μg/mL) for 48 h. **(A)** Representative ORO staining images (scale bar = 100 μm) and quantification of extracted dye absorbance at 520 nm. **(B)** Representative Western blot images and quantitative comparison of SIRT1, p-AMPK, p-ACC, and SREBP-1 levels protein levels, normalized to total AMPK, ACC, and β-actin. **(C)** Representative Western blot images and quantitative comparison of GPR35, PKA, and CREB protein levels, normalized to β-actin. The data represents mean ± SD of three independent experiments. Statistical analysis was performed using one-way ANOVA followed by Tukey’s *post-hoc* test. *p < 0.05, **p < 0.01, and ***p < 0.001 vs. control group; #*p* < 0.05, ##*p* < 0.01 and ###*p* < 0.001 vs. PA group.

Based on the HPLC analysis of Hongjam, silk protein (fibroin) was synthesized into a peptide form for functional validation. We investigated the effects of silk fibroin peptide—the main component identified in Hongjam—using a palmitic acid (PA)-induced MASLD model in HepG2 cells. To assess its impact on lipid accumulation, intracellular lipid content was measured by ORO staining. As expected, PA treatment markedly increased lipid deposition, while silk fibroin significantly reduced lipid accumulation in a dose-dependent manner (Figure 7A). In addition, silk restored the expression of SIRT1 and p-AMPK, and enhanced p-ACC (Figure 7B). In contrast, PA stimulation elevated the expression of SREBP-1, a key lipogenic transcription factor, whereas silk fibroin suppressed its expression in a dose-dependent manner (Figure 7B). We further examined whether the lipid-lowering effect of silk fibroin involves GPR35-mediated signaling. Western blot analysis revealed that silk fibroin upregulated GPR35 expression in a dose-dependent manner (Figure 7C). The expression of downstream effectors PKA and CREB was also elevated (Figure 7C). Taken together, these findings suggest that silk fibroin improves hepatic lipid metabolism, reduces intracellular lipid accumulation, and activates GPR35 signaling, supporting its potential contribution to the hepatoprotective effects of Hongjam in PA-induced MASLD.

## 4 Discussion

MASLD is closely linked to metabolic syndrome, insulin resistance, type 2 diabetes, and obesity, with obesity being a significant contributor to metabolic syndrome and a key factor in MASLD development (3). HFD consumption has been shown to promote hepatic lipid accumulation (29). MASLD occurs due to an imbalance between triglyceride (TG) synthesis and FFA oxidation in hepatocytes (30). In the liver, SIRT1/AMPK signaling pathway is a key regulatory system for lipid metabolism (30). SIRT1 is one of the seven mammalian homologs of Sir2, which catalyzed NAD + -dependent protein deacetylation (31). SIRT1 regulates apoptosis and DNA repair, and it senses NAD + levels to modulate cellular metabolism (32). Indeed, SIRT1 knock-out mice exhibit obesity, insulin resistance, hepatic lipid accumulation, and inflammation (33). Furthermore, SIRT1 is an upstream regulator of AMPK, a crucial cellular energy sensor, and it controls TG synthesis and FFA oxidation (34). SIRT1/AMPK has been reported to be suppressed in MASLD patients. It has been reported that inhibition of SIRT1/AMPK reduces fatty acid oxidation and increases the expression of genes related to triglyceride synthesis in liver tissue (13). Recent studies have further demonstrated that mTOR acts as a central hub integrating nutrient sensing and metabolic signaling (35). Dysregulation of mTOR signaling has been implicated in metabolic disorders such as diabetes and obesity (35). mTOR coordinates transcriptional programs that regulate cellular growth, lipid metabolism, and energy balance, responding to changes in nutrient and amino acid availability (35, 36). In the present study, Hongjam treatment enhanced SIRT1 expression and activated downstream AMPK signaling, leading to increased phosphorylation of AMPK and ACC and upregulation of CPT1, thereby promoting fatty acid oxidation and reducing lipogenesis. Furthermore, compared with the positive control, silymarin, Hongjam did not affect food intake (Supplementary Figure 1) but effectively restored hepatic lipid metabolism. Collectively, these findings indicate that Hongjam alleviates hepatic steatosis by activating the SIRT1/AMPK signaling pathway, suggesting its potential as a functional food for improving metabolic dysfunction in MASLD.

STAT3 plays a crucial role in the development of liver inflammation and the inhibition of liver damage (37). Various cytokines and growth factors are regulated through the STAT3 pathway (37). STAT3 is primarily regulated by Janus kinase (JAK), where the activation of JAK leads to the phosphorylation of specific tyrosine residues on receptors (8). These phosphorylated tyrosine sites, along with adjacent amino acid sequences, form specific docking sites to which STAT proteins bind (8). Once phosphorylated, the activated STAT proteins translocate to the nucleus to regulate the transcription of target genes (37). While STAT3 controls cell survival and proliferation, its chronic activation can lead to various pathological conditions (8). In the liver, inhibiting STAT3 has been shown to prevent MASLD-induced hepatic fibrosis and protect the liver from lipotoxicity (8). Indeed, liraglutide, a first-line treatment for type 2 diabetes, has been reported to reduce inflammation and HFD-induced MASLD by modulating Kupffer cell M2 polarization through the STAT3 signaling pathway (38). In this study, inflammatory cytokines such as TNF-*α* and IL-1β, which were elevated by HFD, were reduced by Hongjam intake. Additionally, Hongjam decreased p-STAT3 expression and downregulated STAT3 target genes. Notably, these effects were found to be more beneficial than those of silymarin, suggesting that Hongjam effectively inhibits the STAT3 pathway, thereby alleviating liver inflammation.

GPR35, an orphan GPCR, primarily interacts with Gαi/o proteins to regulate adenylyl cyclase, which in turn modulates cAMP levels (3). This regulation is crucial because cAMP plays a significant role in controlling various cellular processes, including the suppression of inflammatory factors. Activation of GPR35 has been shown to reduce weight gain and fat accumulation in metabolic disorders such as obesity and type 2 diabetes, suggesting its relevance to lipid metabolism (3). In recent studies, GPR35 has also been associated with protective effects in the liver. Overexpression of GPR35 in hepatocytes prevented steatohepatitis in models subjected to high-fat/cholesterol/fructose diet, while GPR35 knockout models showed aggravated metabolic disorders and hepatic steatosis (39). The increase in cAMP levels, regulated by GPCRs like GPR35, leads to the activation of PKA, which phosphorylates key proteins involved in cellular functions such as growth, differentiation, and apoptosis (40). Importantly, PKA plays a critical role in lipid metabolism, influencing pathways that prevent lipid accumulation in the liver. For example, resveratrol has been shown to reduce hepatic lipid accumulation via a PKA-dependent mechanism (41). Thus, the regulation of cAMP by GPR35 and its downstream activation of the PKA pathway are crucial for maintaining lipid balance and protecting against metabolic liver diseases like MASLD. In this study, Hongjam increased the expression of GPR35 and PKA, demonstrating more beneficial effects compared to silymarin. This suggests that Hongjam may regulate GPR35 and PKA to maintain lipid homeostasis and potentially prevent metabolic liver diseases such as MASLD.

Gluconeogenesis is the process of converting substrate such as glycerol, lactate, and glucogenic amino acids into glucose, primarily occurring in the liver (42). This process is regulated by various factors, including key enzymes and transcription factors (42). Pyruvate, a major substrate for gluconeogenesis, is converted to oxaloacetate by pyruvate carboxylase and malate dehydrogenase, and then to phosphoenolpyruvate (PEP) by PCK1 (42). The converted PEP undergoes a series of transformations to become fructose-1,6-bisphosphate, then fructose-6-phosphate, and ultimately glucose-6-phosphate (42). Glucose-6-phosphate is transported to the endoplasmic reticulum where it is converted to glucose by G6Pase (42). Key regulators of gluconeogenesis include PKA, PCK1, and G6Pase, which are controlled by various transcription factors such as CREB and FoxO1 (43). Inhibiting CREB transcription activity significantly reduces the expression of PCK1 and G6Pase, leading to decreased glucose production (43). Generally, gluconeogenesis exacerbates conditions such as MASLD and type 2 diabetes by promoting glucose and lipid synthesis. However, gluconeogenesis also plays an essential role in maintaining energy homeostasis under metabolic stress, and its regulation depends on hepatic lipid status. Under conditions of prolonged lipid overload, excessive free fatty acids can impair mitochondrial oxidative function and reduce the availability of gluconeogenic substrates, thereby suppressing hepatic glucose production (44, 45). Accumulated fatty acids and their derivatives increase ROS generation and activate AMPK, which in turn inhibits key transcription factors such as CREB and FOXO1 involved in PCK1 and G6Pase expression (46, 47). Thus, chronic exposure to high levels of FFAs can shift hepatic metabolism from glucose production toward lipid storage and stress adaptation, leading to a reduction in gluconeogenic capacity. In this study, Hongjam increased the expression of CREB and PGC-1α, as well as the gluconeogenesis-related genes G6Pase and PCK1. These effects were found to be more beneficial than those of silymarin, suggesting that Hongjam can effectively restore gluconeogenesis diminished by a HFD.

The stronger efficacy of Hongjam compared with silymarin can be attributed to its multi-component composition and distinct mechanism of action. Unlike silymarin, which primarily exerts antioxidant and anti-inflammatory effects through the inhibition of ROS generation and NF-κB signaling (48, 49), Hongjam contains various bioactive compounds, including amino acids, flavonoids, and unsaturated fatty acids, which collectively enhance its hepatoprotective efficacy (19). In the current study, Hongjam increased the expression of GPR35, a G-protein-coupled receptor involved in energy and glucose metabolism, which further contributes to its hepatoprotective and metabolic effects. In addition to its metabolic efficacy, previous acute and subchronic toxicity studies have reported that oral administration of silkworm powder at a dose of 2 g/kg caused no toxic effects in Wistar rats (50). In our previous study, a repeated oral dose toxicity test was performed at 625,1250, and 2,500 mg/kg/day for 4 and 13 weeks, showing no toxicological abnormalities, and the no observed adverse effect level (NOAEL) was determined to be 2,500 mg/kg/day (51). These results indicate that Hongjam is safe, exhibits no adverse effects, and has potential for use as an edible material or functional food ingredient. In the present study, Hongjam was administered to mice at doses of 0.01 g/kg and 0.1 g/kg, corresponding to approximately 0.049 g/day and 0.487 g/day for a 60 kg adult when calculated using body surface area normalization according to Reagan-Shaw et al. (52). The 12-weeks administration period in mice corresponds to approximately 9 years of a human lifespan, suggesting that continuous intake of Hongjam can help ameliorate hepatic steatosis.

Recent studies have suggested that adopting a high-protein, high-soluble fiber, and low-carbohydrate diet may be beneficial for the treatment of MASLD (53). Silkworms are highly valued as functional materials due to their abundance of diverse bioactive compounds (19). Silkworm powder, known as Hongjam, has been reported to contain various functional substances, including proteins, amino acids, flavonoids, vitamins, unsaturated fatty acids, and essential minerals (19). In the present study, HPLC analysis revealed that Hongjam contained abundant amino acids such as Asp., Thr, Ser, Glu, Gly, Ala, Val, Met, Ile, Leu, Tyr, Phe, Lys, His, and Arg, with Gly, Ala, and Ser being the most predominant (Figure 1). These amino acids are known to constitute silk fibroin, the major protein component of silkworms. Silk fibroin has been reported to exert beneficial effects in preventing type 2 diabetes and metabolic syndrome and has gained attention as a promising biomaterial for biomedical applications (54, 55). In this study, silk fibroin-derived peptides appeared to activate the SIRT1/AMPK signaling pathway, suppress lipogenesis, and enhance GPR35 signaling, thereby contributing to the restoration of lipid and glucose metabolism. Taken together, these findings suggest that silk fibroin may be a key bioactive constituent mediating the metabolic benefits of Hongjam, warranting further *in vivo* studies to validate its mechanistic role. As this study was limited to mouse models and HepG2 cells, clinical studies are necessary to evaluate the translational potential of Hongjam as a functional food for MASLD prevention. Considering its origin as an edible silkworm-derived natural material, Hongjam holds great promise as a functional food ingredient targeting metabolic liver diseases.

## 5 Conclusion

In conclusion, our study demonstrated that Hongjam effectively ameliorates metabolic dysfunction-associated steatotic liver disease by inhibiting lipogenesis, enhancing fatty acid oxidation, and restoring gluconeogenesis. Additionally, the administration of Hongjam significantly suppressed the phosphorylation of STAT3, alleviating liver inflammation. These findings suggest that Hongjam is a novel substance for preventing fatty liver disease and mitigating liver inflammation. Importantly, fibroin peptide, a major silk-derived protein component of Hongjam, also exhibited hepatoprotective effects *in vitro* by modulating the SIRT1/AMPK and GPR35/PKA signaling pathways. These results demonstrate that fibroin peptide may serve as a functional bioactive constituent contributing to the metabolic benefits of Hongjam. Overall, our findings support the potential of Hongjam as a natural agent for preventing fatty liver disease and mitigating liver inflammation. Further studies are warranted to elucidate the detailed mechanisms and confirm the *in vivo* efficacy of the fibroin peptide.

## Data Availability

The original contributions presented in the study are included in the article/Supplementary material, further inquiries can be directed to the corresponding author.

## Supplementary material

SUPPLEMENTARY Figure 1 Effects of Hongjam on food intake in HFD-fed mice. **(A)** Weekly changes in food intake during the 12-week experimental period. **(B)** Average food intake throughout the study. Data are expressed as mean ± SD (*n* = 8). Statistical analysis was performed using one-way 710 ANOVA followed by Tukey’s *post-hoc* test. ****p* < 0.001 vs. normal group.

SUPPLEMENTARY Figure 2 Analytical HPLC chromatogram of the synthesized fibroin-derived peptide. The peptide showed a single major peak at a retention time of 1.425 min, corresponding to a purity of 98.3%. Minor peaks (1.708 min and 6.867 min) accounted for < 2% of total area.
