# Supplementary figures and images for: Processed silkworm powder (Hongjam) ameliorates metabolic dysfunction-associated steatotic liver disease via GPR35/PKA and SIRT1/AMPK pathways
Source: Front Nutr. 2025 Dec 3;12:1727043. doi: 10.3389/fnut.2025.1727043 (PMC12708540; doi:10.3389/fnut.2025.1727043)

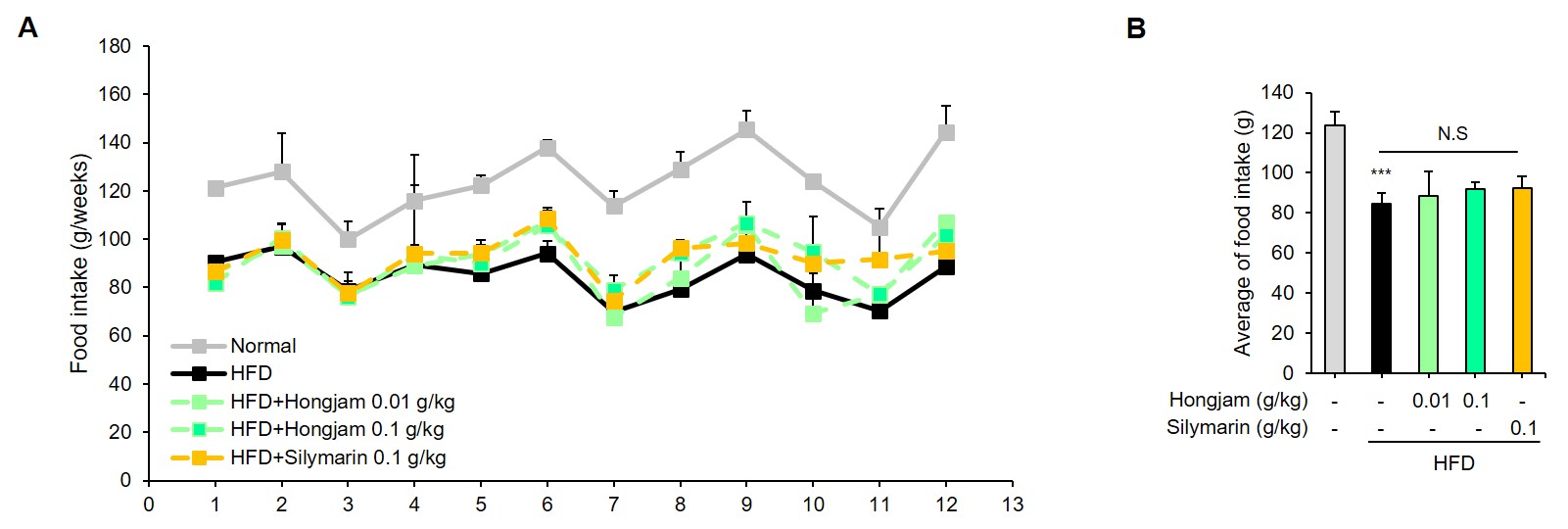

Supplement: SUPPLEMENTARY Figure 1 — Effects of Hongjam on food intake in HFD-fed mice. (A) Weekly changes in food intake during the 12-week experimental period. (B) Average food intake throughout the study. Data are expressed as mean ± SD (n = 8). Statistical analysis was performed using one-way 710 ANOVA followed by Tukey’s post-hoc test. ***p < 0.001 vs. normal group. [file Image_1.JPEG]

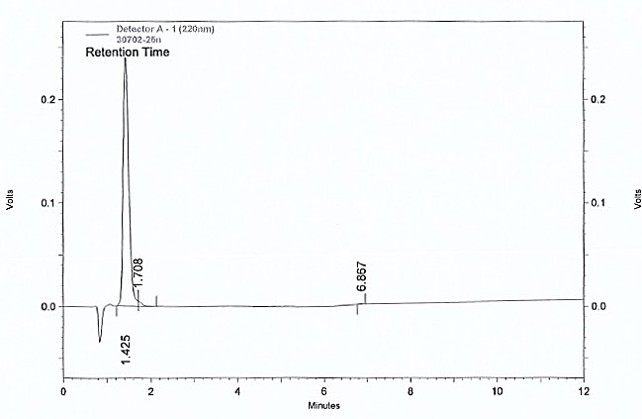

Supplement: SUPPLEMENTARY Figure 2 — Analytical HPLC chromatogram of the synthesized fibroin-derived peptide. The peptide showed a single major peak at a retention time of 1.425 min, corresponding to a purity of 98.3%. Minor peaks (1.708 min and 6.867 min) accounted for < 2% of total area. [file Image_2.JPEG]
