# Supplementary material for: Processed silkworm powder (Hongjam) ameliorates metabolic dysfunction-associated steatotic liver disease via GPR35/PKA and SIRT1/AMPK pathways
Source: Front Nutr. 2025 Dec 3;12:1727043. doi: 10.3389/fnut.2025.1727043 (PMC12708540; doi:10.3389/fnut.2025.1727043)
Supplement: Supplementary file 3 [file Table_1.docx]

**Supplementary table 1.**

| **Content** | **Normal** | **HFD** |
| --- | --- | --- |
| **Protein (kcal%)** | 24 | 20 |
| **Carbohydrate (kcal%)** | 58 | 20 |
| **Fat (kcal%)** | 18 | 60 |
| **Total (kcal%)** | 100 | 100 |

| **Content** | **HFD** | **HFD+ Hongjam 0.01 g/kg** | **HFD + Hongjam 0.1 g/kg** | **HFD + Silymarin 0.1 g/kg** |
| --- | --- | --- | --- | --- |
| **Casein (g)** | 2067.58 | 2067.58 | 2067.58 | 2067.58 |
| **L-cystine (g)** | 31.01 | 31.01 | 31.01 | 31.01 |
| **Maltodextrin (g)** | 1292.24 | 1292.24 | 1292.24 | 1292.24 |
| **Sucrose (g)** | 711.25 | 711.25 | 711.25 | 711.25 |
| **Cellulose (g)** | 516.90 | 516.90 | 516.90 | 516.90 |
| **Soybean Oil (g)** | 258.45 | 258.45 | 258.45 | 258.45 |
| **Lard (g)** | 2532.79 | 2532.79 | 2532.79 | 2532.79 |
| **Mineral mix (g)** | 103.38 | 103.38 | 103.38 | 103.38 |
| **Dicalcium phosphate (g)** | 134.39 | 134.39 | 134.39 | 134.39 |
| **Calcium carbonate (g)** | 56.86 | 56.86 | 56.86 | 56.86 |
| **Potassium citrate (g)** | 170.58 | 170.58 | 170.58 | 170.58 |
| **Vitamin mix (g)** | 103.38 | 103.38 | 103.38 | 103.38 |
| **Choline bitartrate (g)** | 20.68 | 20.68 | 20.68 | 20.68 |
| **Blue dye (g)** | 0.52 | 0.52 | 0.52 | 0.52 |
| **Hongjam (g)** | - | 0.4 | 4 | - |
| **Silymarin (g)** | - | - | - | 4 |
