# Supplementary material for: Processed silkworm powder (Hongjam) ameliorates metabolic dysfunction-associated steatotic liver disease via GPR35/PKA and SIRT1/AMPK pathways
Source: Front Nutr. 2025 Dec 3;12:1727043. doi: 10.3389/fnut.2025.1727043 (PMC12708540; doi:10.3389/fnut.2025.1727043)
Supplement: Supplementary file 4 [file Table_2.docx]

**Supplementary table 2. List of primers**

| **Gene** | **Forward** | **Reverse** |
| --- | --- | --- |
| ***SREBP-1c*** | CTGGTGAGTGGAGGGACCAT | GGTGGATGGGCAGTTTGTCT |
| ***FASN*** | GGAGGTGGTGATAGCCGGTAT | TGGGTAATCCATAGAGCCCAG |
| ***CPT-1*** | GGCATAAACGCAGAGCATTCCTG | CAGTGTCCATCCTCTGAGTAGC |
| ***c-fos*** | GGGAATGGTGAAGACCGTGTCA | GCAGCCATCTTATTCCGTTCCC |
| ***HIF-1α*** | CCTGCACTGAATCAAGAGGTTGC | CCATCAGAAGGACTTGCTGGCT |
| ***c-myc*** | TGCGACGAGGAAGAGAATTT | AACCGCTCCACATACAGTCC |
| ***TNF-α*** | ACTGAACTTCGGGGTGATCG | GCTTGGTGGTTTGCTACGAC |
| ***IL-1β*** | AAATCTCGCAGCAGCACATCAA | CCACGGGAAAGACACAGGTAGC |
| ***IL-6*** | TACCACTTCACAAGTCGGAGGC | CTGCAAGTGCATCATCGTTGTTC |
| ***GPR35*** | CTCTGCTCCTTGCCATTTGTGC | AGCAATGGCAGTGACCAGGCTT |
| ***PKA*** | CAGACTTCGGTTTTGCCAAGCG | GCCATCTCGTAGATGAGGACTC |
| ***CREB*** | CACAGACCACTGGACAGCA | AGGACGCGATAACAACTCCAGG |
| ***PGC-1α*** | GAATCAAGCCACTACAGACACCG | CATCCCTCTTGAGCCTTTCGTG |
| ***G6Pase*** | AGGTCGTGGCTGGAGTCTTGTC | GTAGCAGGTAGAATCCAAGCGC |
| ***PCK1*** | GGCGATGACATTGCCTGGATGA | TGTCTTCACTGAGGTGCCAGGA |
| ***18s rRNA*** | GCAATTATTCCCCATGAACG | GGCCTCACTAAACCATCCAA |
